# Prescribing and Treatment in the Diseases of Infants and Children

**Published:** 1892-06

**Authors:** 


*Prescribing and Treatment in the Diseases of Infants and Children.* By PHILIP E. MUSKETT. Pp. 293. Edinburgh: YOUNG J. PENTLAND. 1891.

This is another of the ready-reference books arranged in dictionary form, which, judging by their number, seem to be in favour with the profession. The author has compiled it from the best known and most trustworthy works on the diseases of children, and the compilation has been well done. The drugs are given first; under each of them there are stated the diseases in which it has been found serviceable, the dose, and mode of administration. Then comes another section, comprising diseases arranged in alphabetical order, with a brief account of the symptoms of each, and the treatment recommended by various authors. The nature of the book necessitates that a good deal must be sacrificed to brevity. Often the reader will run the chance of being bewildered by the list of remedies advocated for a given disease, without sufficient indication as to which one is likely to give the best results for his particular needs. The danger of using such books—although they are undoubtedly useful in many ways, and this is a good one of the sort—lies in the temptation they afford to shirk the labour of getting a sound knowledge of disease by systematic clinical observation and reading, and to substitute for it a reference to a dictionary of treatment to see "what is good for so-and-so."

